# Potentially Inappropriate Prescribing and Potential Prescribing Omissions and Their Association with Adverse Drug Reaction-Related Hospital Admissions

**DOI:** 10.3390/jcm13020323

**Published:** 2024-01-06

**Authors:** Ross Brannigan, John E. Hughes, Frank Moriarty, Emma Wallace, Ciara Kirke, David Williams, Kathleen Bennett, Caitriona Cahir

**Affiliations:** 1 School of Population Health, RCSI University of Medicine and Health Sciences, D02 YN77 Dublin, Ireland; branniganross@rcsi.ie (R.B.); kathleenebennett@rcsi.ie (K.B.); 2 School of Pharmacy and Biomolecular Sciences, RCSI University of Medicine and Health Sciences, D02 YN77 Dublin, Ireland; frankmoriarty@rcsi.ie; 3 Department of General Practice, University College Cork, T12 R229 Cork, Ireland; ewallace@ucc.ie; 4 National Quality and Patient Safety Directorate at Health Service Executive, D08 W2A8 Dublin, Ireland; ciara.kirke@hse.ie; 5 Department of Geriatric and Stroke Medicine, RCSI University of Medicine and Health Sciences, D02 YN77 Dublin, Ireland; davidwilliams@rcsi.ie; 6 Department of Geriatric and Stroke Medicine Beaumont Hospital, D05 E840 Dublin, Ireland

**Keywords:** potentially inappropriate prescribing, potential prescribing omissions, adverse drug reactions, hospital admissions, older populations

## Abstract

Background: This study aimed to determine the prevalence of potentially inappropriate prescribing (PIP) and potential prescribing omissions (PPOs) and their association with ADR-related hospital admissions in patients aged ≥ 65 years admitted acutely to the hospital. Methods: Information on medications and morbidities was extracted from the Adverse Drug Reactions in an Ageing Population (ADAPT) cohort (N = 798: N = 361 ADR-related admissions; 437 non-ADR-related admissions). PIP and PPOs were assessed using Beers Criteria 2019 and STOPP/START version 2. Multivariable logistic regression (adjusted odds ratios (aOR), 95%CI) was used to examine the association between PIP, PPOs and ADR-related admissions, adjusting for covariates (age, gender, comorbidity, polypharmacy). Results: In total, 715 (90%; 95% CI 87–92%) patients had ≥1 Beers Criteria, 555 (70%; 95% CI 66–73%) had ≥ 1 STOPP criteria and 666 patients (83%; 95% CI 81–86%) had ≥ 1 START criteria. Being prescribed at least one Beers (aOR = 1.66, 95% CI = 1.00–2.77), or meeting STOPP (aOR = 1.07, 95% CI = 0.79–1.45) or START (aOR = 0.72; 95%CI = 0.50–1.06) criteria or the number of PIP/PPO criteria met was not significantly associated with ADR-related admissions. Patients prescribed certain drug classes (e.g., antiplatelet agents, diuretics) per individual PIP criteria were more likely to have an ADR-related admission. Conclusion: There was a high prevalence of PIP and PPOs in this cohort but no association with ADR-related admissions.

## 1. Introduction

In 2017, the World Health Organisation (WHO) put forward medication safety as their third Global Patient Safety Challenge, creating a framework that allows for the reduction of severe, avoidable medication-related harm by 50% globally over the next 5 years [1]. Older populations (aged ≥ 65 years) are at a greater risk of medication-related harm due to increased multimorbidity and medication utilisation and a variety of physiological changes affecting the pharmacokinetics and pharmacodynamics of medications [2]. It is estimated that adverse drug reactions (ADRs; noxious and unintended responses to medicinal products) account for approximately 10–20% of hospital admissions in older populations [3,4].

Potentially inappropriate prescribing (PIP) involves prescribing medications with a greater risk than benefit to patients, and potential prescribing omissions (PPOs) involve not prescribing medications of potential benefit to patients [5]. Prevalent in older populations, there are numerous reasons why a medication might be considered potentially inappropriate, including drug–drug or drug–disease interactions, safer alternative medications, increased drug toxicity affecting kidney function, or the potential exacerbation of ongoing chronic conditions [6]. The American Geriatrics Society (AGS) Beers Criteria and the European Screening Tool of Older Persons’ Potentially Inappropriate Prescriptions (STOPP) and the Screening Tool to Alert Doctors to the Right Treatment (START) are the most frequently used and validated measures of PIP and PPOs, respectively [7]. The Beers Criteria were originally developed in the United States in 1991 and updated in 1997, 2003, 2012, 2015 and 2019 and have recently been updated for 2023 [8,9]. The STOPP/START criteria were originally developed in 2008, revised in 2015 and recently updated in 2023 [6,10].

A 2015 systematic review of the prevalence and type of PIP in twenty-three different European countries of community-dwelling older populations estimated an overall PIP prevalence of 22.6% (CI 19.2–26.7%; range 0–98%), with 10 studies using the Beers Criteria (1997 and 2003 versions), 14 studies using the STOPP criteria (2008 version) and eight studies using the START criteria (2008 version) [11]. To date, few studies have examined the prevalence of PIP and PPOs among community-dwelling older adults using both the revised STOPP/START version 2 [6] and 2019 Beers Criteria [8] or compared them [12].

PIP and PPO criteria are clinically relevant if they can significantly reduce the rate of ADRs or other forms of medication-related harm in older populations. Reviews have estimated that approximately 6–12% of hospital admissions in older populations are due to ADR-related factors, with PIPs and PPOs contributing to between 7% and 17% of ADR-related hospital admissions [13,14]. One study investigating the association between the STOPP (2008 version) and occurrence of ADRs considered PIPs to be the cause of 60% of ADRs affecting the vascular system, 50% of ADRs affecting the nervous system and 62.5% of ADRs resulting in falls [15], while being prescribed the appropriate START medication has been associated with a reduction in mortality [16]. However, to date, there is a lack of research assessing the predictive validity of STOPP/START version 2 and the Beers 2019 and how they are associated with ADRs and medication-related harm in older populations.

The aim of this study is to determine the prevalence of PIP and PPOs using the recently updated STOPP/START version 2 and 2019 Beers Criteria and their association with ADR-related hospital admissions in patients aged ≥ 65 years admitted acutely to hospital in Ireland.

## 2. Methods

### 2.1. Study Design

This study used the Adverse Drug Reactions in an Ageing Population (ADAPT) cohort (N = 798), a cross-sectional and prospective cohort study designed to examine the prevalence and risk factors for ADR-related hospital admissions, in all patients aged ≥65 years admitted acutely to a large tertiary referral hospital in Ireland over an 8-month period (November 2016–June 2017) [17,18]. Ethical approval was obtained from the Beaumont Hospital Ethics Committee (REC 16/49).

### 2.2. ADR-Related Hospital Admissions

Within the ADAPT cohort, 3091 patients were screened upon hospital admission, and 361 (11.7% 95% CI 10.5%, 12.8%) patients had an ADR-related admission [18]. ADR-related admissions were determined using a multifaceted review of each hospital admission to assess the likelihood of the ADR being a reason for admission (cause of admission or contributing to admission) in the context of the patient’s medication, clinical conditions, medical history, comorbidities and investigations and using validated algorithms and decision aids [19,20]. A sample of patients who were determined not to have a suspected ADR at hospital admission were randomly assigned to a non-ADR hospital admission control group from the hospital admission list, which detailed patients’ chronological order of hospital admission on each day for those aged ≥ 65 years (N = 437) [17,18].

### 2.3. Exposure to PIP and PPOs

The 2019 Beers Criteria [8] and the STOPP version 2 criteria [6] were applied to the ADAPT cohort. The 2019 Beers Criteria consists of 6 different tables of PIP criteria, and all were applied to the cohort (Supplementary Beers Tables S2–S7 The first of the tables, Beers Table S2, includes medications that are potentially inappropriate in most older adults. Beers Table S3 includes medications that are potentially inappropriate in older adults due to drug–disease or drug–syndrome interactions that may exacerbate the disease or syndrome. Beers Table S4 lists medications to be used with caution in older adults. Beers Table S5 lists potentially clinically important drug–drug interactions that should be avoided in older adults. Beers Table S6 lists medications that should be avoided or have their dosage reduced with varying levels of kidney function, and Beers Table S7 lists medications with strong anticholinergic properties. In addition to Beers Table S7, other strong anticholinergic medications known to have an adverse impact on older adults as per the Drug Burden Index (DBI) were included [21].

The STOPP version 2 criteria include 80 PIP criteria based on physiological systems, with additional categories relating to patients at risk of falls, patients taking opioid analgesics and patients taking drugs with anticholinergic properties. PPOs were identified using the START criteria, which consist of 34 criteria, also arranged according to physiological systems, and include more important and common instances of potentially beneficial medication that may be inappropriately omitted. Similar to previous research, not all Beers 2019 and STOPP/START version 2 criteria were applied due to lack of information within ADAPT on (1) clinical test results; (2) severity of disease status; (3) duration of medication use; and (4) information on the rank ordering of first choice medications [22]. All of the Beers 2019, except two, and the 48 (60%) STOPP and 23 (68%) START criteria were applied (Supplementary Beers Tables S2–S7, Supplementary STOPP version 2 Table, Supplementary START version 2 Table).

For each patient, details on their medications (ATC codes), primary presenting complaint and co-morbidities at hospital admission were extracted. The primary presenting complaint and other co-morbidities were coded using the Medical Dictionary for Regulatory Activities (MedDRA) terminology [23]. Details of any clinical investigations, including creatinine clearance (CrCl) calculated using the Cockcroft–Gault equation, were also extracted (N = 614; 77%), as well as information on patients’ functional and cognitive abilities and any impairments. Within ADAPT, patients self-reported if they were immobile (Yes/No), their level of mobility (use of walking aids when crossing a room and when outside), if they had a functional impairment and their falls history (fallen previously, fallen in the last year, fallen more than once). Frailty was assessed using the Triage Risk Screening Tool and the PRISMA-7 [24]. Delirium was assessed using the 4AT [25] and DSM4 criteria [26]. The application of all PIP and PPO criteria to the ADAPT cohort was reviewed by a pharmacist (JEH) and a consultant clinical pharmacologist (DW).

### 2.4. Covariates

Covariates included age, sex (female vs. male), comorbidity and polypharmacy, which are known risk factors associated with ADR-related hospital admissions [2,11,18]. Comorbidity was measured using the Charlson co-morbidity score (Charlson weights 0, 1 and 2, ≥3) [27], and polypharmacy was measured as none (≤ 4 medications), polypharmacy (5 to 9 medications) and significant polypharmacy (≥ 10 medications) [28].

### 2.5. Data Analysis 

#### 2.5.1. PIP and PPO Prevalence

The overall PIP and PPO prevalence per the Beers 2019 and STOPP/START version 2 criteria was calculated as (i) the proportion of patients with at least one PIP/PPO; (ii) the proportion of patients with either 1, 2, or ≥ 3 PIPs/PPOs; and (iii) the average (median, IQR) number of PIP/PPO criteria per patient. The prevalence for the individual Beers 2019 and STOPP/START version 2 criteria was also calculated (Supplementary Beers Tables S2–S7, Supplementary STOPP version 2 Table, Supplementary START version 2 Table). In the case of the individual Beers 2019 or STOPP/START version 2 criteria, which specified a medical condition/presence of disease or medication, prevalence was calculated based on the number of patients with the specified medical condition/presence of disease or medication.

#### 2.5.2. PIP and PPOs and ADR-Related Hospital Admissions

Univariate and multivariate logistic regression was used to examine the association between the presence of at least one PIP/PPO and the number of PIP/PPOs (1, 2, ≥3 criteria) and ADR-related hospital admissions for each of the three sets of criteria (Beers 2019, STOPP/START version 2). Unadjusted odds ratios (OR) and adjusted odds ratios (aOR) for covariates and 95% confidence intervals (CI) are reported. Models were adjusted for age, sex, co-morbidity and polypharmacy. The association between individual PIP and PPO criteria was also examined using univariate logistic regression, with Bonferroni corrections for multiple testing. Data analysis and application of the PIP/PPO criteria to the data set was performed using Stata 17 (StataCorp, Texas, USA).

## 3. Results

### 3.1. Study Population

The mean age of the ADAPT cohort (N = 798) was 80.85 (SD = 7.56), with 256 (32%) patients aged over 85 years and 417 (52%) who were female. In total, 324 (41%) patients had a Charlson Comorbidity Index score ≥ 3, 313 (39.2%) experienced significant polypharmacy and 441 (55%) were determined to be frail as per the Triage Risk Screening Tool [29]. Patients with an ADR-related hospital admission were significantly younger than those without an ADR-related hospital (mean age 80 versus 82, respectively) [18]. There were no statistically significant differences in gender, co-morbidity and polypharmacy between those with an ADR-related hospital admission and those without [18].

### 3.2. Overall PIP and PPO Prevalence and Number of Criteria per Patient

In total, 741 patients (93%; 95% CI 91–94) were prescribed at least one Beers 2019 or STOPP version 2 criteria. Within the 7% (N = 56) of patients who were not prescribed a PIP indicator, 39 had a PPO per the START version 2. Overall, 780 patients (98%; 95% CI 96–99) had at least one PIP or PPO upon hospital admission (median = 5, IQR 3–8)

When combining all 2019 Beers Criteria across the six tables, 715 (90%) patients had at least one criterion, with almost half of the patients having three or more criteria. A lower proportion of patients (N = 555; 70%) had at least one STOPP (48/80 criteria applied) and one START version 2 criteria (N = 666; 83%) (23/34 criteria applied) (Table 1).

### 3.3. Prevalence of PIP According to Individual 2019 Beers Criteria

In total, 309 (39%; 95% CI 35–42) ADAPT patients had at least one PIP from Beers Table S2 (medications that are potentially inappropriate in most older adults), with the most frequent criteria being the use of zolpidem (N = 62 (8%); 95% CI 6–10). There were 198 (25%; 95% CI 22–28) patients with at least one PIP per Beers Table S3 (PIP due to drug–disease or drug–syndrome interactions), with the use of corticosteroids in the presence of delirium being the most prevalent criteria (85/205 with delirium; 42%; 95% CI 35–48). There were 670 patients (84%; 95% CI 81–86) with at least one PIP per Beers Table S4 (medications to be used with caution in older adults). The most common was the use of diuretics (N = 450 patients; 56%; 95% CI 53–60) and aspirin (N = 420 patients; 53%; 95% CI 49–56). There were 183 patients (77%; 95% CI 74–80) patients with a PIP per Beers Table S5 (drug–drug interactions that should be avoided in older adults), with any combination of three or more CNS-active drugs (N = 114; 14% 95% CI 12–17) being the most prevalent criterion. There were 100 (13%; 95% CI 10–15) patients with at least one PIP criterion per Beers Table S6 (medications that should be avoided or have their dosage reduced with varying levels of kidney function), with pregabalin in those with creatinine clearance <60 (N = 403 with creatinine clearance <60) (42/403; 10% 95% CI 8–14) having the highest prevalence. There were 120 (15%; 95% CI 13–18) patients with at least one PIP per Beers Table S7 (medications with strong anticholinergic properties), with the use of amitriptyline (N = 33; 4%; 95% CI 3–6) having the highest prevalence (Supplementary Beers Tables S2–S7).

### 3.4. Prevalence of PIP According to Individual STOPP Version 2 Criteria

The most prevalent STOPP version 2 PIP criterion was the use of loop diuretics as a treatment for hypertension (N = 159; 20% 95% CI 17–23), although it was unknown if it was first-line treatment. This was followed by 156 patients prescribed hypnotic Z-drugs, (20%; 95% CI 17–22). Approximately 10% of patients were prescribed a benzodiazepine; with a similar prevalence found forantiplatelet agents with a vitamin K antagonist, direct thrombin inhibitor or factor Xa inhibitors with stable coronary, cerebrovascular or peripheral arterial disease and antimuscarinic drugs with dementia or narrow-angle glaucoma or chronic prostatism (Supplementary STOPP version 2 Table).

### 3.5. Prevalence of PPOs According to Individual START Version 2 Criteria

The most prevalent PPO was lack of bisphosphonates and vitamin D and calcium in patients taking long-term systemic corticosteroid therapy (N = 56/62; 90% 95% CI 80–96), though the duration of corticosteroid therapy use was unknown. This was followed by the absence of a non-TCA antidepressant in patients with depression (N = 56/77; 73% 95% CI 62–82). An angiotensin-converting enzyme (ACE) inhibitor was also absent for 422 patients who had systolic heart failure and/or documented coronary artery disease (N = 618; 68% 95% CI 65–72) (Supplementary START version 2 Table).

### 3.6. PIP and PPOs and ADR-Related Hospital Admissions

Table 2 presents the associations between the different PIP and PPO criteria and whether the hospital admission was ADR-related or non-ADR-related.

Having at least one Beers 2019 PIP was significantly associated with an admission being ADR-related (*p* = 0.04), but after covariate adjustment (age, gender, morbidity and polypharmacy), the association was no longer significant (*p* = 0.05). The number of 2019 Beers Criteria was also not significantly associated with an admission being ADR-related. There was no association with having at least one STOPP or START version 2 criterion or the number of STOPP version 2 criteria and ADR-related hospital admissions. Having three or more START version 2 criteria was shown to be significantly associated with a lower likelihood of an admission being ADR-related (*p* = 0.04), but the association was no longer significant after covariate adjustment.

Patients prescribed Beers Criteria from Table S4 (e.g., diuretics, aspirin) were almost twice as likely to have a hospital admission that was ADR-related (OR = 1.94; 95% CI 1.30, 2.90; *p* < 0.01) (Supplementary Beers Tables S2–S7; Table S8). The use of antiplatelet agents, alongside other agents in coronary, cerebrovascular and peripheral arterial disease, as per STOPP version 2 criteria, was also significantly associated with an admission being ADR-related (OR= 2.96 95% CI 1.81, 4.85; *p* < 0.01). (Supplementary STOPP version 2 Table).

## 4. Discussion

The overall prevalence of PIP and PPOs in this cohort of older people admitted to the hospital using the updated 2019 Beers Criteria (715; 90%) and STOPP (555; 70%) and START (666; 83%) version 2 criteria was high. A recent study of PIP in older patients in an intensive care unit reported a PIP prevalence of 80.6% per the 2019 Beers Criteria and 59.7% per STOPP version 2 [30], while a study of community-based patients reported a prevalence of 68.8% per the 2019 Beers Criteria and 57.4% per STOPP version 2 [31]. Studies of PPOs in older populations using START version 2 criteria have reported prevalence rates of 79% in the inpatient setting [32] and 67% in the community setting [22]. The higher prevalence of PIP and PPOs in the current study could be due to the advanced age (mean age of 81 years) of the cohort, compared to previous studies, and the higher level of polypharmacy (≥ 10 medications; 39.2% vs. 17.9%) and morbidity burden [31]. Increasing age, multimorbidity and polypharmacy are well-known risk factors for PIP and PPOs [33].

The PIP and PPO prevalence rates identified in the current study with the updated criteria are also higher than previously reported rates in Ireland using the 2012 Beers Criteria (42%) [34] and STOPP/START version 1 (52.7% and 38.2%, respectively) [35]. The STOPP/START version 2 includes a 31% increase in prescribing criteria compared to version 1 (80 vs. 65 STOPP criteria; 34 vs. 22 START criteria) and has been shown to be more sensitive (higher prevalence) [6,36], while the updated 2019 Beers Criteria include new medications and the addition of selected drug–drug interactions [8]. The most prevalent PIP criteria per the Beers 2019 and STOPP version 2 included the use of and combinations of hypnotic drugs (Z-drugs), anxiolytics (especially benzodiazepines), antipsychotics and antidepressants where interaction, potentiation or reduction in therapeutic efficacy can occur, while PPOs included a lack of bisphosphonates, calcium, vitamin D and ACE inhibitors where these were indicated. Similar findings have been reported in previous studies [32,36]. The PPO of depression not treated with drug therapy has also been previously identified in Ireland, with two thirds of older people with depression not prescribed antidepressant therapy [37].

This is the first large-scale study to investigate the association between PIP and PPOs and ADR-related hospital admissions using the revised Beers 2019 and STOPP/START version 2 criteria. No statistically significant association was determined after covariate adjustment. Previous research using STOPP/START version 2 reported a significantly higher proportion of drug-related admissions (DRAs) in older patients compared with STOPP/START version 1, particularly admissions related to falls and fractures [32]. An increased risk of mortality and hospitalisation in the community setting has also being reported using START version 2 but not for STOPP version 2 [22]. The current study did identify a statistically significant association between individual PIP criteria and ADR-related hospital admissions, including the use of aspirin and diuretics. A prospective review of 6,427 cases of ADRs recorded in German Pharmacovigilance Centers in those aged ≥ 70 years identified two to four causative drugs (e.g., aspirin), and intake of particular compounds (e.g., spironolactone) but not the total numbers of medications or PIP as risk factors for ADRs [38].

## 5. Strengths and Limitations

This study is one of the first large-scale studies on ADR-related hospital admissions and PIP/PPOs in Ireland. A gold-standard medication reconciliation list was used to determine the prevalence of PIP and PPOs, where the patient’s medication list was verified by a pharmacist against two alternative sources [39]. The ADAPT cohort also contains detailed information on clinical investigations, including creatinine clearance, morbidity and functional and cognitive abilities, enabling the application of a large number of PIP and PPO criteria [17]. The determination of an ADR-related hospital admission included a multifaceted review of each suspected ADR, including clinical judgement and chart review, and the application of a number of validated algorithms by two investigators based on standard criteria [18]. Nearly all consecutive hospitalisations in older people due to acute illnesses were included, thereby reducing selection bias.

However, there are some limitations to consider. The study cohort included older, frail patients with multiple comorbidities who were prescribed on average 10 or more medications admitted acutely to the hospital. The control group included patients admitted acutely to the hospital but where the admission was determined not to be ADR-related. The results may, therefore, not be generalisable to other settings or to the general older population. A number of STOPP (approximately 40%) and START version 2 criteria (approximately 33%) were not applied due to insufficient information within the ADAPT cohort. In general, the STOPP/START version 2 criteria require a level of detail that may not be available in standard medical records (e.g., bone mineral density T-scores, estimated glomerular filtration rate).

## 6. Implications

There is a lack of reliable and valid ADR detection and prediction tools developed for use in community settings, and predictive factors for ADR-related hospital admissions are still poorly understood [18]. While PIP and PPO criteria are informative, the lists of criteria are extensive and are limited by their single drug/disease-orientated approach. Further research needs to be undertaken to understand the complex interplay between higher-risk drugs and drug classes, multimorbidity and frailty and how they result in ADR-related hospital admissions [40].

There are indications that using PIP and PPO criteria as a screening tool may result in lower prevalence of ADRs, although, to date, findings are not statistically significant [41]. Further research is ongoing using longitudinal cohort studies. Gallagher et al., (2011) found that using the START/STOPP as a screening tool and making recommendations to physicians improved prescription appropriateness and significantly reduced the risk of unnecessary polypharmacy, incorrect dosage and DDIs [42]. In Sweden, pharmacists identified PIP and PPOs with patients and their families and significantly reduced the number of PIP and PPOs and drug-related re-admissions by 80% after one year [43,44]. Appropriate deprescribing and medication reviews/optimisation have been identified as interventions that lower the risk of ADR-related admissions in older populations [45]. However medication reviews/optimisation in clinical practice is challenging, and multiple barriers may need to be overcome, including increasing physician knowledge on how best to balance medication benefits and harms in complex older multimorbid polypharmacy patients, engaging patients and their families/carers and having adequate time and resources [46,47].

## 7. Conclusions

The prevalence of PIP and PPOs within the ADAPT cohort was higher than what has previously been reported in other hospitalised cohorts. There were no statistically significant associations between the Beers 2019 and STOPP/START version 2 criteria and ADR-related hospital admissions compared to non-ADR-related admissions. However, there was an indication that certain drug classes (e.g., antiplatelet agents, diuretics) as per individual PIP criteria are significantly associated with ADR-related hospital admissions. A greater focus on establishing the ADR risk associated with particular drugs and drug classes in multimorbid older frail patients may be more informative.

## Figures and Tables

**Table 1:** The number (%) of patients with PIP/PPO and the median (IQR) number of PIP/PPO criteria per patient (N = 798).

	Number (%) of Patients with PIP and PPO				
Criteria	At least 1 PIP/PPO	1	2	≥3	Median (IQR) PIP/PPO
Beers 2019	715 (90) 95% CI (87–92)	167 (21) 95% CI (18–24)	159 (20) 95% CI (17–23)	389 (49) 95% CI (45–52)	2 (1, 3)
STOPP version 2	555 (70) 95% CI (66–73)	212 (27) 95% CI (24–30)	152 (19) 95% CI (16–22)	191 (24) 95% CI (21–27)	1 (0, 2)
START version 2	666 (83) 95% CI (81–86)	161 (20) 95% CI (17–23)	192 (24) 95% CI (21–27)	313 (39) 95% CI (36–43)	2 (1, 3)

PIP = Potentially inappropriate prescribing, PPO= Potential prescribing omissions.

**Table 2:** Number (percentage), unadjusted and adjusted odds ratios (95% CI) for patients with an ADR-related hospital admission compared to a non-ADR related hospital admission by exposure to PIP and PPO.

	ADR-Related Hospital Admission (N = 361) N (%)	Non-ADR Related Hospital Admission (N = 437) N (%)	Unadjusted Odds Ratio (95% CI)	Adjusted Odds Ratio (95% CI) *
At least one Beers 2019 criterion vs. none	332 (92.0)	383 (87.6)	1.61 (1.00, 2.59) *	1.66 (0.99, 2.76)
At least one STOPP v.2 criterion vs. none	254 (70.4)	301 (68.9)	1.07 (0.79, 1.45)	1.01 (0.73, 1.41)
At least one START v.2 criterion vs. none	292 (80.9)	374 (85.6)	0.72 (0.49, 1.04)	0.71 (0.48, 1.04)
*Number of Beers 2019 criteria*				
0 (reference)	29 (8.0)	54 (12.4)	-	-
1	76 (21.1)	91 (20.8)	1.56 (0.90, 2.68)	1.61 (0.93, 2.81)
2	74 (20.5)	85 (19.5)	1.62 (0.94, 2.81)	1.67 (0.93, 2.99)
≥3	182 (50.4)	207 (47.4)	1.64 (1.00, 2.68)	1.71 (0.97, 3.00)
*Number of STOPP v.2 criteria*				
0 (reference)	107 (29.6)	136 (31.1)	-	-
1	92 (25.5)	120 (27.5)	0.97 (0.67, 1.41)	0.94 (0.64, 1.38)
2	71 (19.7)	81 (18.5)	1.11 (0.74, 1.67)	1.06 (0.68, 1.64)
≥3	91 (25.2)	100 (22.9)	1.16 (0.79, 1.69)	1.11 (0.72, 1.70)
*Number of START v.2 criteria*				
0 (reference)	69 (19.1)	63 (14.4)	-	-
1	73 (20.2)	88 (20.1)	0.76 (0.48, 1.20)	0.72 (0.45, 1.16)
2	89 (24.7)	103 (23.6)	0.79 (0.51, 1.23)	0.76 (0.48, 1.20)
≥3	130 (36.0)	183 (41.9)	0.65 (0.43, 0.98) *	0.66 (0.43, 1.02)

Covariates include age, sex, co-morbidity and polypharmacy. * *p* < 0.05. PIP = Potentially inappropriate prescribing, PPO = Potential prescribing omissions, ADR = Adverse drug reaction.

## Data Availability

The dataset presented in this article is not publicly available for use. Requests to access the dataset should be directed to caitrionacahir@rcsi.ie.

## Supplementary Materials

The following supporting information on the Beers 2019 Supplementary Tables S2–S7, Table S8, Supplementary STOPP version 2 Table, Supplementary START version 2 Table can be downloaded at: https://www.mdpi.com/article/10.3390/jcm13020323/s1.
