# Supplementary material for: Potentially Inappropriate Prescribing and Potential Prescribing Omissions and Their Association with Adverse Drug Reaction-Related Hospital Admissions
Source: J Clin Med. 2024 Jan 6;13(2):323. doi: 10.3390/jcm13020323 (PMC10816937; doi:10.3390/jcm13020323)
Supplement: Supplementary file 1 [file jcm-13-00323-s001.zip › jcm-2782210-supplementary.pdf]

START Version 2 Supplementary Table

Table S1: Medications that are potentially omitted in older populations (N=798)

Criteria	N (%) Disorder	N (%) PPO	95% CI	ADR OR (95% CI)	Omission/Limitations
Bisphosphonates and vitamin D and calcium in patients taking long-term systemic corticosteroid therapy.	62 (7.8)	56 (90)	80- 96	0.94 (0.14-6.11)	Unknown duration of corticosteroid therapy
Non-TCA antidepressant drug in the presence of persistent major depressive symptoms.	77 (9.6)	56 (72.7)	62 –82	1.82 (0.59-5.69)	Unknown duration of symptoms. Applied to those with a diagnosis of depression
Angiotensin Converting Enzyme (ACE) inhibitor with systolic heart failure and/or documented coronary artery disease.	618 (77.4)	422 (68.3)	65 -72	0.69 (0.49-0.98; p=0.04)	-
Acetylcholinesterase inhibitor (e.g. donepezil, rivastigmine, galantamine) for mild-moderate Alzheimer’s dementia or Lewy Body dementia (rivastigmine).	109 (13.7)	73 (67)	58 -75	0.51 (0.22-1.18)	Could not establish severity of Alzheimer’s dementia or Lewy body dementia
Disease-modifying anti-rheumatic drug (DMARD) with active, disabling rheumatoid disease.	28 (3.5)	18 (64.3)	44- 80	2.37 (0.36-15.49)	Could not establish whether or not rheumatoid disease was disabling
Bone anti-resorptive or anabolic therapy (e.g. bisphosphonate, strontium ranelate, teriparatide, denosumab) in patients with documented osteoporosis, where no pharmacological or clinical status contraindication	92 (11.5)	52 (56.5)	46 - 66	3.25 (1.20-8.78; p=0.02)	Could not establish bone density scores

Criteria	N (%) Disorder	N (%) PPO	95% CI	ADR OR (95% CI)	Omission/Limitations
exists (Bone Mineral Density T-scores -> 2.5 in multiple sites) and/or previous history of fragility fracture(s).					
Statin therapy with a documented history of coronary, cerebral or peripheral vascular disease, unless the patient's status is end-of-life or age is > 85 years.	637 (79.8)	312 (49)	45 -53	0.32 (0.90-1.95)	-
Laxatives in patients receiving opioids regularly.	87 (10.9)	43 (49.4)	39 -60	0.98 (0.39-2.51)	-
Regular inhaled corticosteroid for moderate-severe asthma or COPD, where FEV1 <50% of predicted value and repeated exacerbations requiring treatment with oral corticosteroids.	224 (28.1)	107 (47.8)	41-54	0.89 (0.50-1.58)	Could not establish if FEV1 <50% of predicted value
ACE inhibitor or Angiotensin Receptor Blocker (if intolerant of ACE inhibitor) in diabetes with evidence of renal disease i.e. dipstick proteinuria or microalbuminuria (>30mg/24 hours) with or without serum biochemical renal impairment.	60 (7.5)	29 (48.3)	36-61	0.80 (0.24-2.61)	-
Vitamin D and calcium supplement in patients with known osteoporosis and/or previous fragility fracture(s) and/or (Bone Mineral Density T-scores more than -2.5 in multiple sites).	92 (11.5)	38 (41.3)	32 -52	0.69 (0.29-1.64)	Cannot establish fragility fractures or bone density scores

Criteria	N (%) Disorder	N (%) PPO	95% CI	ADR OR (95% CI)	Omission/Limitations
Beta-blocker with ischaemic heart disease.	353 (44.2)	140 (39.7)	34 -45	0.76 (0.49-1.18)	-
Antiplatelet therapy (aspirin or clopidogrel or prasugrel or ticagrelor) with a documented history of coronary, cerebral or peripheral vascular disease.	637 (79.8)	242 (38)	34 - 42	0.65 (0.47-0.92; p=0.01)	-
Appropriate beta-blocker (bisoprolol, nebivolol, metoprolol or carvedilol) with stable systolic heart failure.	159 (19.9)	52 (32.7)	26- 40	0.79 (0.39-1.62)	-
Xanthine-oxidase inhibitors (e.g. allopurinol, febuxostat) with a history of recurrent episodes of gout.	41 (5.1)	13 (31.7)	19 -48	1.29 (0.26-6.30)	-
Selective serotonin reuptake inhibitor (or SNRI or pregabalin if SSRI contraindicated) for persistent severe anxiety that interferes with independent functioning.	23 (2.9)	7 (30.4)	15 -53	10.01 (0.23-430.62)	Could not establish if anxiety interferes with independent functioning
Vitamin D supplement in older people who are housebound or experiencing falls or with osteopenia (Bone Mineral Density T-score is > -1.0 but < -2.5 in multiple sites).	145 (18.2)	44 (30.3)	23 -38	0.98 (0.44-2.17)	Could not establish bone density scores
Topical prostaglandin, prostamide or beta-blocker for primary open-angle glaucoma	22 (2.8)	6 (27.3)	12 -50	23.21 (0.37-1427.18)	Could not establish if open-angle glaucoma, just presence of glaucoma
Vitamin K antagonists or direct thrombin inhibitors or factor Xa inhibitors in	46 (5.8)	9 (19.6)	10 - 34	0.20 (0.03-1.42)	-

Criteria	N (%) Disorder	N (%) PPO	95% CI	ADR OR (95% CI)	Omission/Limitations
the presence of chronic atrial fibrillation.					
Proton Pump Inhibitor with severe gastro-oesophageal reflux disease or peptic stricture requiring dilatation.	30 (3.8)	6 (20)	9- 39	1.77 (0.19-16.69)	Could not establish severity

* Using Bonferroni corrections for 20 criteria applied p< 0.003; no criteria were significantly associated with ADR-related hospital admissions

Beers 2019 Supplementary Tables S2-S7

Table S2: Medications that are potentially inappropriate in older populations (N=798)

Medication/Medication Class	N (%)	95% CI	Limitations
Zolpidem	62 (7.77)	6.10-9.84	
Antipsychotics (1st & 2nd generation)	57 (7.02)	5.34-9.02	Could not establish if used as an antiemetic during chemotherapy
Amitriptyline	33 (4.13)	2.86-5.76	
Diazepam	30 (3.76)	2.55-5.32	
Digoxin	28 (3.51)	2.34-5.03	Included if A.FIB or heart failure were present
Doxazosin	21 (2.63)	1.64-3.99	Included if medication and hypertension present
Belladonna alkaloids	17 (2.13)	1.25-3.39	
Ibuprofen	15 (1.88)	1.06-3.08	
Metoclopramide	11 (1.38)	0.69-2.45	
Flurazepam	11 (1.38)	0.69-2.45	
Temazepam	10 (1.25)	0.60-2.29	
Triazolam	9 (1.13)	0.50-2.13	
Amiodarone	9 (1.13)	0.50-2.13	Counted if A.FIB or Heart Failure were present. Could not establish substantial left ventricular hypertrophy
Naproxen	8 (1.00)	0.43-1.97	
Lorazepam	8 (1.00)	0.43-1.97	
Promethazine	6 (0.75)	0.28-1.63	
Nitrofurantoin	6 (0.75)	0.28-1.63	

Medication/Medication Class	N (%)	95% CI	Limitations
Ketoprofen	6 (0.75)	0.28-1.63	
Atropine	6 (0.75)	0.28-1.63	
Diclofenac	5 (0.63)	0.20-1.46	Chronic use not determined
Clonazepam	5 (0.63)	0.20-1.46	

Potentially identifiable data where n<5 are not presented**.**

Excluded criteria: Oestrogen, Reserpine

Table S1: Potentially inappropriate medication use in older adults due to drug-disease or drug-syndrome interactions that may exacerbate the disease or syndrome

Medication/Medication Class	N (proportion)	95% CI	Limitations/ Notes
Syncope (N=67)			
Participants with syncope prescribed at least one PIPs	9 (0.13)	0.07 – 0.24	
Delirium (N=205)			
Anticholinergics	56 (0.27)	0.22 – 0.34	
Antipsychotics/Benzodiazepines	33 (0.16)	0.12 – 0.22	
Corticosteroids	85 (0.42)	0.35 – 0.48	
Zolpidem	15 (0.07)	0.04 – 0.12	
Participants with delirium prescribed at least one of above PIPs	124 (0.61)	0.54 – 0.67	
Dementia or Cognitive Impairment (N=258)			
Anticholinergics	72 (0.28)	0.23 – 0.34	
Antipsychotics/Benzodiazepines	61 (0.24)	0.19 – 0.29	
Zolpidem	17 (0.07)	0.04- 0.10	
Participants with dementia/Cognitive impairment prescribed at least one of above PIPs	107 (0.41)	0.36 – 0.48	
History of falls/fracture (N=67)			
Antiepileptics	186 (0.24)	0.15 – 0.38	
Antipsychotics	8 (0.12)	0.06 – 0.22	
Benzodiazepines	5 (0.07)	0.03 – 0.17	

Medication/Medication Class	N (proportion)	95% CI	Limitations/ Notes
Zolpidem	7 (0.10)	0.05 – 0.21	
Antidepressants	23 (0.34)	0.24 – 0.46	
SNRIs	5 (0.07)	0.03 – 0.17	
Opioids	9 (0.10)	0.05 – 0.21	
Participants with history of falls/fracture prescribed at least one of above PIPs	47 (0.70)	0.58 – 0.80	

Potentially identifiable data where n<5 are not presented

N (proportion) of patients per condition or disease group

Table S4: Medications to be used with caution in older adults (N=798)

Medication/Medication Class	N (%)	95% CI	Limitations/Notes
Diuretics	450 (56.39)	52.87-59.87	
Aspirin	420 (52.63)	49.10-56.14	
SSRI	115 (14.41)	12.05-17.04	
SNRI	76 (9.52)	7.58 – 11.78	
Antipsychotics	71 (8.89)	7.01-11.09	
Mirtazapine	39 (4.89)	3.50-6.62	
TCA	39 (4.89)	3.50-6.62	
Rivaroxaban	38 (4.76)	3.39-6.48	
Tramadol	38 (4.76)	3.39-6.48	
Dabigatran	9 (1.13)	0.52-2.13	
Prasugrel	5 (0.62)	0.20-1.46	

Potentially identifiable data where n<5 are not presented

Table S5: Drug-drug interactions that should be avoided in older adults (N=798)

Medication/Medication Class	N (%)	95% CI	Limitations/Notes
Three or more: Antidepressants, Antipsychotics, Antiepileptics, Opioids, Benzodiazepines and Hypnotic Z-drugs	114 (14.29)	11.93 – 16.91	
Peripheral α-1 blockers + Loop diuretics	41 (5.14)	3.71 – 6.91	

Opioids + Gabapentin/Pregabalin	21 (2.63)	1.64 – 3.99	
Warfarin + Amiodarone	14 (1.75)	0.96 - 2.93	
RAS inhibitor/potassium-sparing diuretics + Another RAS inhibitor	13 (1.63)	0.87 – 2.77	
Opioids + Benzodiazepines	10 (1.25)	0.60 – 2.29	
Anticholinergic + Anticholinergic	9 (1.13)	0.52 – 2.13	

Potentially identifiable data where n<5 are not presented

Table S6: Medications that should be avoided or have their dosage reduced with varying levels of kidney function in older adults

Medication/Medication Class	Creatinine Clearance mL/min (N)	N (%)	95% CI	Limitations/Notes
Pregabalin	< 60 (403)	42 (10.42)	0.08 – 0.14	
Rivaroxaban	< 50 (328)	25 (7.62)	0.05 – 0.11	
Levetiracetam	≤ 80 (514)	16 (3.11)	0.02 – 0.05	
Ranitidine	< 50 (328)	12 (3.66)	0.02 – 0.06	
Spironolactone	< 30 (123)	8 (6.50)	0.03 – 0.13	
Gabapentin	< 60 (403)	7 (1.73)	0.01 – 0.04	
Tramadol	< 30 (123)	7 (5.69)	0.03 – 0.12	
Apixaban	< 25 (82)	6 (7.32)	0.03 – 0.16	

Potentially identifiable data where n<5 are not presented

Creatinine clearance (CrCl) calculated using the Cockcroft-Gault equation

N (%) of patients with the specified creatinine clearance and the medication

Table S7: Medications with strong anticholinergic properties (N=798)

Medication/Medication Class	N (%)	95% CI	Limitations/Notes
Amitriptyline	33 (4.1)	2.9-5.8	
Solifenacin	21 (2.6)	1.6-4.0	
Prochlorperazine	18 (2.3)	1.3- 3.5	
Tolterodine	15 (1.9)	1.1-3.1	
Promethazine (antihistamines)	6 (0.8)	0.2-1.6	
Fesoterodine	6 (0.8)	0.2-1.6	
Olanzapine	6 (0.8)	0.2-1.6	
Atropine	6 (0.8)	0.2-1.6	

Potentially identifiable data where n<5 are not presented

Table S8: The association between Beers 2019 criteria Tables S2-S7 and ADR-related hospital admissions

Beers 2019 criteria	Unadjusted odds ratio (95% CI)
Table S2	0.88 (0.66, 1.18)
Table S3	0.98 (0.71, 1.36)
Table S4	1.94 (1.30, 2.90; p=0.001)*
Table S5	1.16 (0.83, 1.62)
Table S6	1.30 (0.87, 1.94)
Table S7	0.66 (0.44, 0.98)

* p<0.05

STOPP Version 2 Supplementary Table

Table S1: Medications that are potentially inappropriate in older populations (N=798)

Criteria	N (%)	95% CI	ADR OR (95% CI)	Omission/Limitations
Loop diuretic as first-line treatment for hypertension (lack of outcome data for this indication; safer, more effective alternatives available).	159 (20)<br><br>Hypertension (N=558; 28.5)	17-23	1.66 (1.15-2.39)	Unable to establish first line treatment
Hypnotic Z-drugs e.g. zopiclone, zolpidem, zaleplon (may cause protracted daytime sedation, ataxia).	156 (19.6)	17-22	0.96 (0.66-1.37)	-
Benzodiazepines (sedative, may cause reduced sensorium, impair balance).	86 (10.8)	9-13	0.74 (0.46 – 1.19)	-
Antiplatelet agents with vitamin K antagonist, direct thrombin inhibitor or factor Xa inhibitors in patients with stable coronary, cerebrovascular or peripheral arterial disease without a clear indication for anticoagulant therapy (no added benefit from dual therapy).	84 (10.5)<br><br>Coronary, cerebrovascular or peripheral arterial disease (N=481; 17.5)	9-13	**2.96 (1.81-4.85; p<0.001)***	Cannot establish without a clear indication for anticoagulant therapy

Criteria	N (%)	95% CI	ADR OR (95% CI)	Omission/Limitations
Antimuscarinic drugs with dementia, or chronic cognitive impairment (risk of increased confusion, agitation) or narrow-angle glaucoma (risk of acute exacerbation of glaucoma), or chronic prostatism (risk of urinary retention).	80 (10)<br><br>Dementia (N=124; 64.5)<br><br>Glaucoma (N=22; 27.5)<br><br>Prostatism (N=65; 81.3)	8-12	1.14 (0.71 – 1.84)	Cannot establish narrow-angle glaucoma
Concomitant use of two or more drugs with antimuscarinic/anticholinergic properties (e.g. bladder antispasmodics, intestinal antispasmodics, tricyclic antidepressants, first generation antihistamines) (risk of increased antimuscarinic/anticholinergic toxicity)	74 (9.3)	7-11	0.55 (0.33 – 0.91; p=0.02)	-
Neuroleptic drugs (may cause gait dyspraxia, Parkinsonism).	71 (8.9)	7-11	0.83 (0.50 – 1.37)	-
Anticholinergics/antimuscarinics in patients with delirium or dementia (N=251) (risk of exacerbation of cognitive impairment).	67 (8.4)<br><br>Delirium/ dementia (N=251; 26.7)		1.06 (0.63-1.80)	-
Anticholinergics/antimuscarinics to treat extra-pyramidal side-effects of neuroleptic medications (risk of anticholinergic toxicity)	60 (7.5)	5.9 -9.6	1.09 (0.64-1.88)	Can only account for the two medications types being taken simultaneously
Non-selective beta-blocker (whether oral or topical for glaucoma) with a history of asthma requiring treatment (risk of increased bronchospasm).	59 (7.4)	6-9	0.77 (0.45-1.34)	Cannot establish asthma requiring treatment

Criteria	N (%)	95% CI	ADR OR (95% CI)	Omission/Limitations
NSAID with severe hypertension (risk of exacerbation of hypertension) or severe heart failure (risk of exacerbation of heart failure).	35 (4.4)<br><br>Hypertension (N=558; 6.2)		0.50 (0.24-1.04)	-
Use of regular (as distinct from PRN) opioids without concomitant laxative (risk of severe constipation).	38 (4.8)	3-6	1.02 (0.53 – 1.97)	-
Any duplicate drug class prescription e.g. two concurrent NSAIDs, SSRIs, loop diuretics, ACE inhibitors, anticoagulants (optimisation of monotherapy within a single drug class should be observed prior to considering a new agent).	31 (3.9)	3-5	2.30 (1.08-5.13)	
Neuroleptic antipsychotic in patients with behavioural and psychological symptoms of dementia (BPSD) unless symptoms are severe and other treatments have failed (increased risk of stroke).	31 (3.9)<br><br>Dementia (N=124; 25)		0.73 (0.35-1.56)	Cannot establish "unless symptoms are severe and other treatments have failed"
Phosphodiesterase type-5 inhibitors (e.g. sildenafil, tadalafil, vardenafil) in severe heart failure characterised by hypotension i.e. systolic BP < 90 mmHg, or concurrent daily nitrate therapy for angina (risk of cardiovascular collapse)	30 (3.8)<br><br>Severe Heart failure (N=145; 20.7)<br><br>Hypotension (N=58; 51.7)	2.6- 5.3	0.88 (0.42-1.87)	Examined concurrent severe heart failure and hypotension
Phenothiazines as first-line treatment, since safer and more efficacious alternatives exist (phenothiazines are sedative, have significant anti-muscarinic toxicity in older people, with the exception of prochlorperazine for nausea/vomiting/vertigo,	21 (2.6)	1.7-4	0.91 (0.37-2.20)	Unable to establish first line treatment

Criteria	N (%)	95% CI	ADR OR (95% CI)	Omission/Limitations
chlorpromazine for relief of persistent hiccoughs and levomepromazine as an anti-emetic in palliative care).				
Acetylcholinesterase inhibitors with a known history of persistent bradycardia (< 60 beats/min.), heart block or recurrent unexplained syncope or concurrent treatment with drugs that reduce heart rate such as beta-blockers, digoxin, diltiazem, verapamil (risk of cardiac conduction failure, syncope and injury).	19 (2)	1.5 - 4	1.53 (0.61-3.86)	Cannot establish persistent bradycardia
Selective alpha-1 selective alpha blockers in those with symptomatic orthostatic hypotension or micturition syncope (risk of precipitating recurrent syncope)	19 (2.4)<br><br>Orthostatic hypotension (N=24;79.2)<br><br>Syncope (N=67;28.4)	1.5-4	2.10 (0.80-5.49)	-
Vitamin K antagonist, direct thrombin inhibitor or factor Xa inhibitors for first pulmonary embolus (N=33) without continuing provoking risk factors for > 12 months (no proven added benefit).	15 (1.9)<br><br>Pulmonary embolus (N=33; 45.5)		4.65 (1.29 – 16.73; 0.019)*	Cannot establish "without continuing provoking risk factors for > 12"
Aspirin with a past history of peptic ulcer disease without concomitant PPI (risk of recurrent peptic ulcer).	14 (1.8)	1-3	3.86 (1.19-12.53)	-
Oral bisphosphonates in patients with a current or recent history of upper gastrointestinal disease i.e. dysphagia, oesophagitis, gastritis, duodenitis, or peptic ulcer disease, or upper gastrointestinal bleeding	14 (1.8)<br><br>Gastrointestinal disease (N=174; 8)		1.52 (0.51-4.48)	-

Criteria	N (%)	95% CI	ADR OR (95% CI)	Omission/Limitations
(risk of relapse/exacerbation of oesophagitis, oesophageal ulcer, oesophageal stricture)				
Aspirin in combination with vitamin K antagonist, direct thrombin inhibitor or factor Xa inhibitors in patients with chronic atrial fibrillation (AF) without a clear indication for aspirin (no added benefit from aspirin)	13 (1.6)<br><br>Chronic AF (N=46; 28.6)		2.85 (0.86-9.42)	Cannot establish without a clear indication for aspirin
Corticosteroids (other than periodic intra-articular injections for mono-articular pain) for osteoarthritis (risk of systemic corticosteroid side-effects).	12 (1.5)<br><br>Osteoarthritis (N=122; 9.8)		3.50 (0.93-13.18)	-
Systemic corticosteroids instead of inhaled corticosteroids for maintenance therapy in moderate-severe COPD (unnecessary exposure to long-term side-effects of systemic corticosteroids and effective inhaled therapies are available).	12 (1.5)<br><br>COPD (N=173; 6.9)		0.58 (0.17-1.98)	-
First-generation antihistamines (safer, less toxic antihistamines now widely available).	11 (1.4)	0.8-2.5	0.92 (0.27-3.07)	-
COX-2 selective NSAIDs with concurrent cardiovascular disease (increased risk of myocardial infarction and stroke)	10 (1.3)<br><br>Cardiovascular disease (N=610; 1.6)		0.46 (0.12-1.81)	-
Drugs likely to cause constipation (e.g. antimuscarinic/anticholinergic drugs, oral iron, opioids, verapamil, aluminium antacids) in patients with chronic constipation (N=13) where non-constipating	9 (1.1)<br><br>Chronic constipation (N=13; 69.2)		1.63 (0.42-6.20)	Cannot establish whether non-constipating alternatives are available

Criteria	N (%)	95% CI	ADR OR (95% CI)	Omission/Limitations
alternatives are available (risk of exacerbation of constipation).				
Tricyclic antidepressants with dementia, narrow angle glaucoma, cardiac conduction abnormalities, prostatism, or prior history of urinary retention (risk of worsening these conditions).	7 (0.9)	0.4 – 1.8	1.58 (0.35-7.21)	Can only establish presence of glaucoma, not whether it is narrow angle.
Vasodilator drugs (e.g. alpha-1 receptor blockers, calcium channel blockers, long-acting nitrates, ACE inhibitors, angiotensin I receptor blockers) with persistent postural hypotension i.e. recurrent drop in systolic blood pressure $\geq$ 20mmHg (risk of syncope, falls).	5 (0.6)<br><br>Postural hypotension (N=24; 20.8)		4.86 (0.53 – 44.53)	-

Potentially identifiable data where n<5 are not presented**.**

*Using Bonferroni corrections for 30 criteria p<0.002
